# Supplementary material for: Visuomotor flexibility is embedded in the topography of frontal cortex
Source: bioRxiv. 2026 May 28:2026.05.25.727601. Preprint. [Version 1] doi: 10.64898/2026.05.25.727601 (PMC13232317; doi:10.64898/2026.05.25.727601)
Supplement: Supplement 1 [file NIHPP2026.05.25.727601v1-supplement-1.pdf]

bioRxiv preprint doi: https://doi.org/10.64898/2026.05.25.727601; this version posted May 28, 2026. The copyright holder for this preprint (which was not certified by peer review) is the author/funder, who has granted bioRxiv a license to display the preprint in perpetuity. It is made available under a CC-BY 4.0 International license.

504

Supplemental Figures

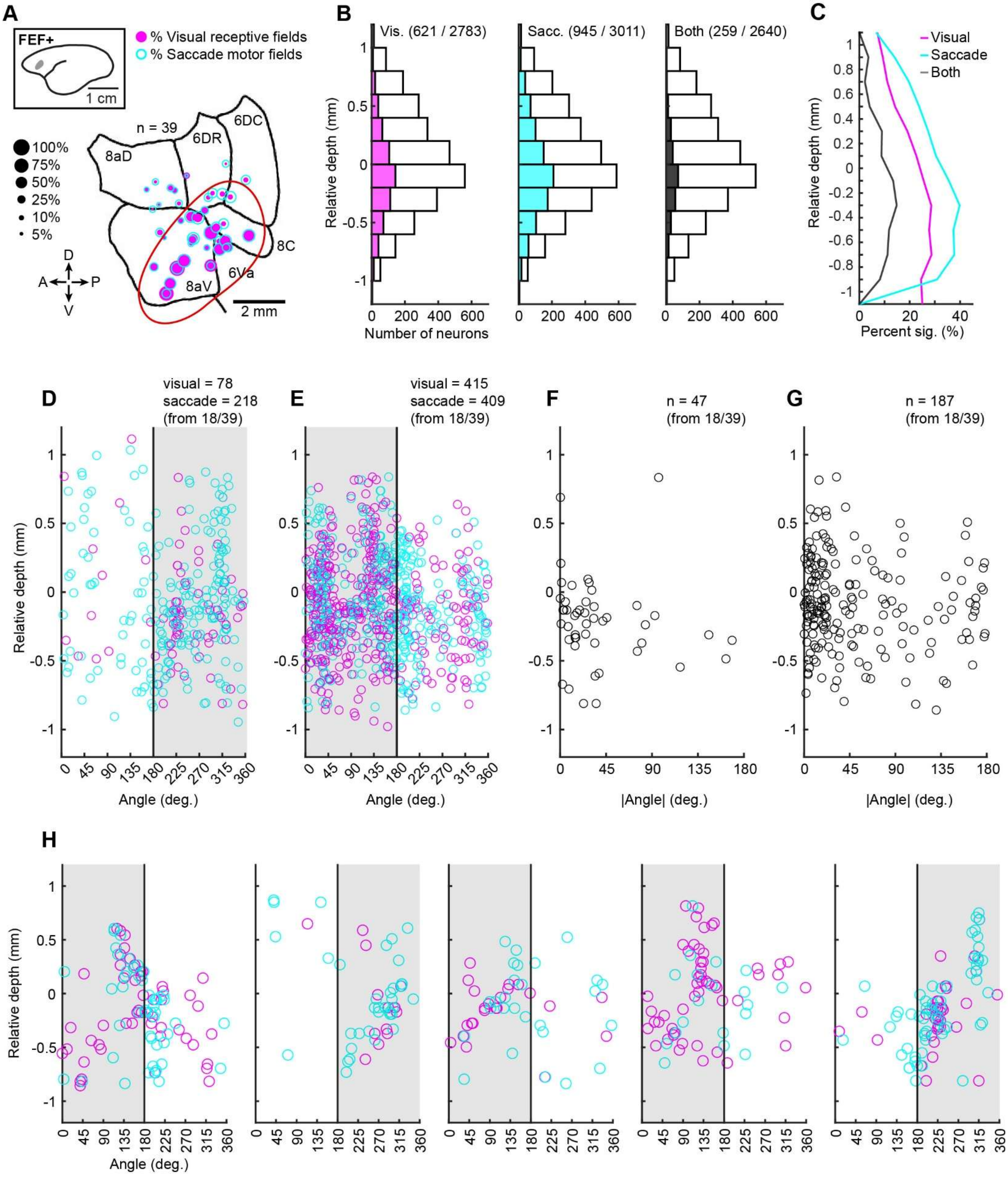

bioRxiv preprint doi: https://doi.org/10.64898/2026.05.25.727601; this version posted May 28, 2026. The copyright holder for this preprint (which was not certified by peer review) is the author/funder, who has granted bioRxiv a license to display the preprint in perpetuity. It is made available under a CC-BY 4.0 International license.

Supp. Fig. 1. Estimated locations of recording sites and summary of RF and MF incidence and vector angles by relative cortical depth. A. Incidence of visual RFs (magenta) and saccade MFs (cyan) shown at the estimated locations of recording sites. Data is combined across both monkeys. Recordings in Monkey F and Monkey H were in the left and right hemispheres, respectively. The red oval indicates the estimated FEF+ region. **B.** Incidence of visual RFs, saccade MFs, and both as a function of depth. White bars indicate the total number of neurons. The inclusion criterion (>2Hz firing rate) is applied separately to each analysis; as a result, the total number of neurons differs across analyses. **C.** Percentage of neurons with RFs, MFs, and both as a function of depth (same data as B). **D, E.** Distribution of RF and MF angles by relative depth for Monkey F (D) and Monkey H (E). Angles are defined with 0° (360°) at the top and increase counterclockwise so that 180° is at the bottom. Under this convention, 0°-180° corresponds to the left hemifield, whereas 180°-360° corresponds to the right hemifield. Shaded region indicates the contralateral hemifield. **F, G**. Distribution of the absolute angle differences between RFs and MFs for neurons with both, distributed by relative depth for Monkey F (F) and Monkey H (G). Only data for recording sites with at least 10 RFs and 10 MFs (18/39) are included for plots D-G. **H.** Examples of the RF and MF angles by relative depth for single penetrations. The first three panels correspond to the examples in Figure 2 E-G. Panels 1, 3, and 4 are from Monkey H, and the remaining panels are from Monkey F. The shaded regions correspond to the contralateral hemisphere.

bioRxiv preprint doi: https://doi.org/10.64898/2026.05.25.727601; this version posted May 28, 2026. The copyright holder for this preprint (which was not certified by peer review) is the author/funder, who has granted bioRxiv a license to display the preprint in perpetuity. It is made available under a CC-BY 4.0 International license.

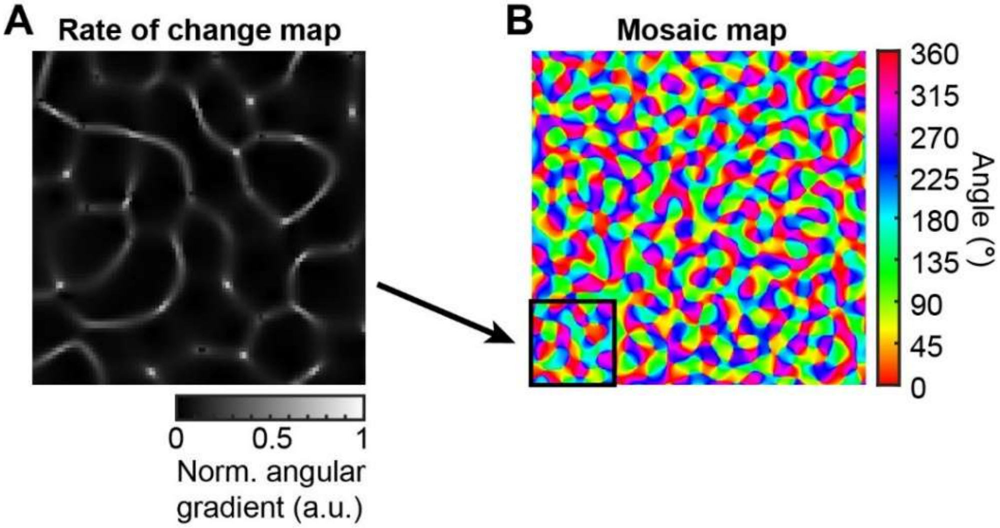

Supp. Fig. 2. The AM model generates a biologically realistic rate of change map (i.e., Weliky et al., 1996). A. Normalized angular gradient computed from a portion of an example mosaic map (shown in **B**). The gradient magnitude quantifies the local rate of change in preferred angle across cortical space. Regions of low gradient reflect smooth, continuous variation in angular preference, whereas high-gradient regions (white curvilinear contours) mark abrupt transitions in angle, analogous to fracture lines.

bioRxiv preprint doi: https://doi.org/10.64898/2026.05.25.727601; this version posted May 28, 2026. The copyright holder for this preprint (which was not certified by peer review) is the author/funder, who has granted bioRxiv a license to display the preprint in perpetuity. It is made available under a CC-BY 4.0 International license.

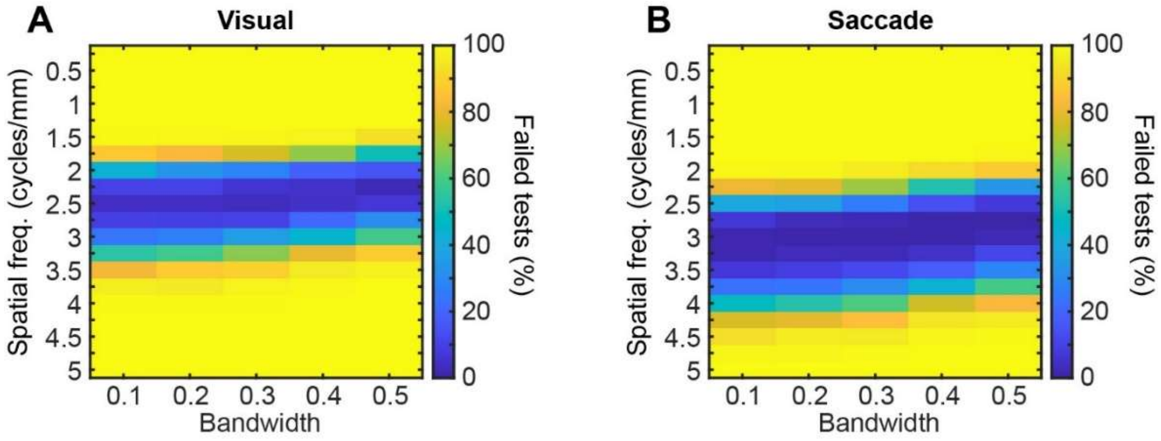

Supp. Fig. 3. Bandwidth had a weak influence on the preferred spatial frequencies. We systematically varied the bandwidth parameter of the AM model to assess its influence on the inferred spatial scale of the mosaic organization. Across a broad range of bandwidth values, model fits were highly stable for both the visual (**A**) and saccade (**B**) maps. Although increasing bandwidth slightly reduced the preferred spatial frequency for both modalities, this effect was weak. Thus, the dominant factor governing the model fits is the central spatial frequency, with bandwidth exerting only a secondary influence. This robustness indicates that the inferred spatial structure of the visual and saccade maps reflects genuine features of the data rather than fine-tuned parameter choices.

bioRxiv preprint doi: https://doi.org/10.64898/2026.05.25.727601; this version posted May 28, 2026. The copyright holder for this preprint (which was not certified by peer review) is the author/funder, who has granted bioRxiv a license to display the preprint in perpetuity. It is made available under a CC-BY 4.0 International license.

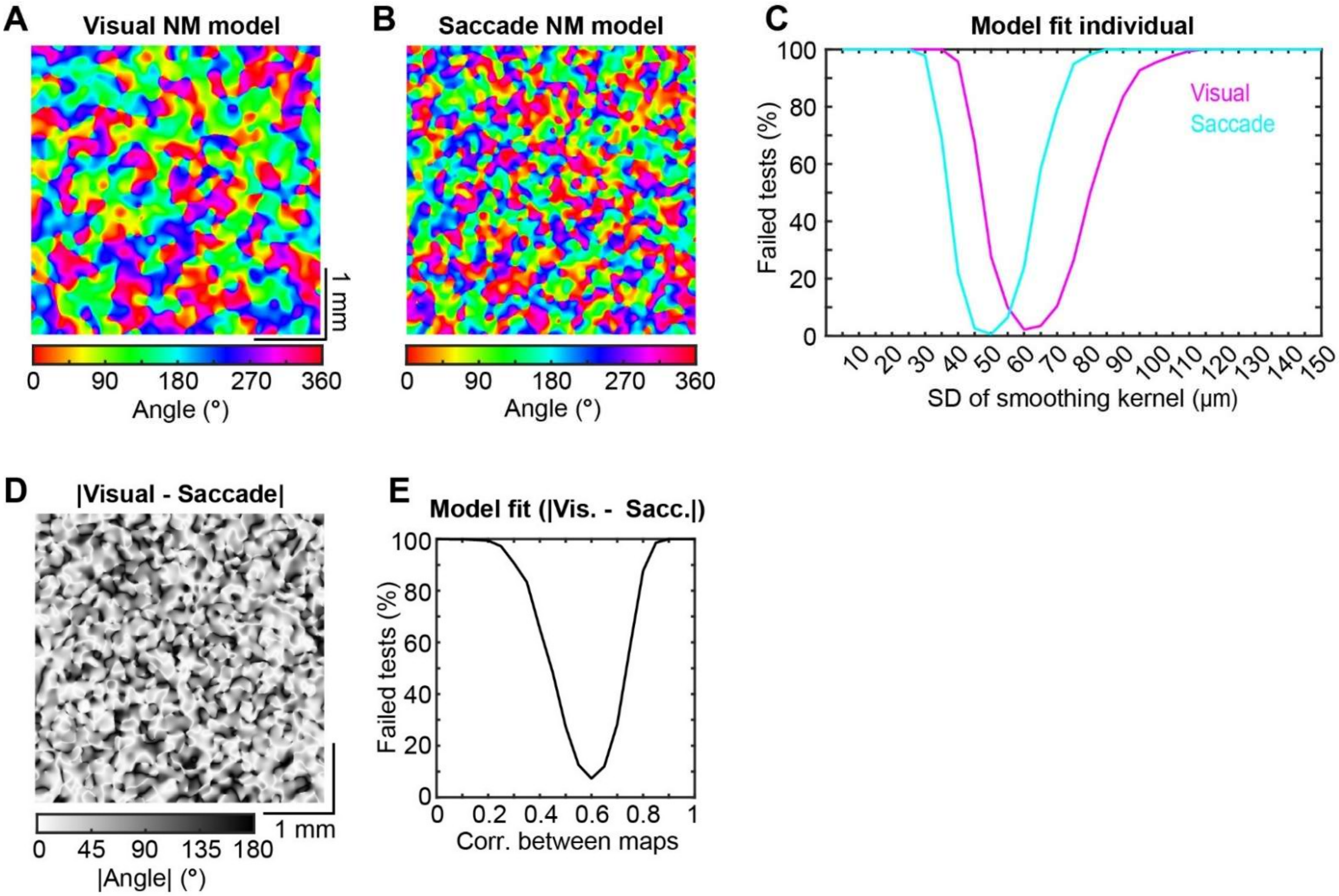

Supp. Fig. 4. NM models replicates real data with partially correlated maps at different preferred spatial scales. A, B. Examples of randomly generated visual (SD = 50 µm) and saccade (60 SD = 60 µm) mosaic maps from the NM model. The example maps are generated with a correlation of 0.6. **C.** Results from testing the distribution of differences from the model to the real data at a range SD values. Only models within a narrow but offset range of SDs fit the data. **D.** Interference pattern obtained by subtracting the example visual and saccade maps (A, B). Despite the absence of periodic structure in the NM model, subtracting partially correlated maps with offset spatial scales produces a structured moiré-like pattern of alternating alignment and divergence. **E.** Model performance as a function of inter-map correlations for NM models with SD = 50 µm and SD = 60 µm. Uncorrelated and fully correlated maps failed to produce the differences between visual and saccade angles, whereas models with a correlation near 0.6 matched the data.

bioRxiv preprint doi: https://doi.org/10.64898/2026.05.25.727601; this version posted May 28, 2026. The copyright holder for this preprint (which was not certified by peer review) is the author/funder, who has granted bioRxiv a license to display the preprint in perpetuity. It is made available under a CC-BY 4.0 International license.

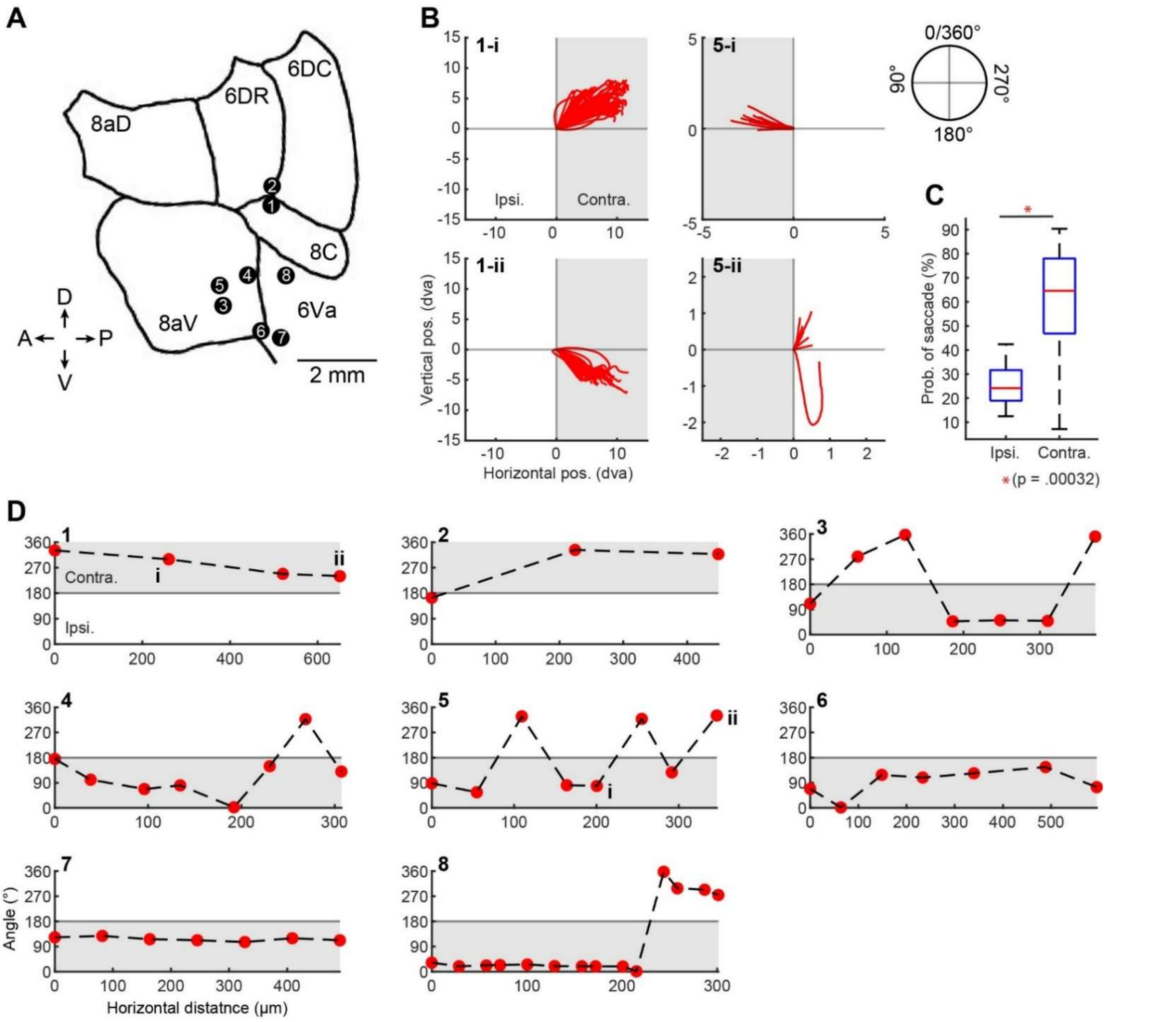

Supp. Fig. 5. The angles of saccades driven by microstimulation are also consistent with a mosaic topography. We observe a similar pattern of smooth changes and abrupt jumps, consistent with a mosaic topography, and prior reports of microstimulation in macaques (Bruce et al., 1985). **A)** Estimated locations of penetration sites. Numbers indicate the associated plots in part D. **B)** Examples of saccades caused by microstimulation at different sites on two different recording sessions. The first column shows saccades being driven to the upper and lower contra-lateral hemifield. The second column shows saccades being driven to the contra and ipsilateral hemifields. The numbers match the plot number in part D, and the roman numerals indicate the site location. **C)** We found that the efficacy of saccade production was lower for the ipsilateral than the contralateral hemifield (Wilcoxon ranked sum test). **D)** Saccade angles for all microstimulation sessions. Red dots indicate the stimulation locations. Gray shading indicates the contralateral hemisphere. Plots 1 and 2 are from Monkey F where a single microelectrode was used, and the rest are from Monkey H where a 64-channel laminar probe was used.

594

bioRxiv preprint doi: https://doi.org/10.64898/2026.05.25.727601; this version posted May 28, 2026. The copyright holder for this preprint (which was not certified by peer review) is the author/funder, who has granted bioRxiv a license to display the preprint in perpetuity. It is made available under a CC-BY 4.0 International license.

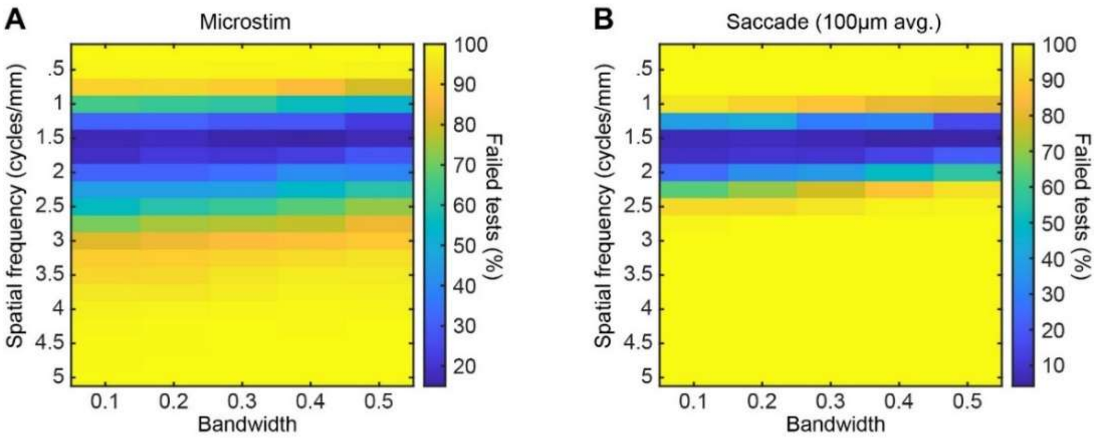

Supp. Fig. 6. Results for mosaic map analysis on microstimulation data and saccade data with a larger spatial average. Using the same analysis applied to the RFs and MFs, we found that only a narrow range of spatial frequencies fit (A). However, the preferred SF range was lower than the preferred SF of the saccade MF angles. This could occur if the stimulation region is larger than the region used to measure the saccade angles, so we increased the region over which the saccade angles were averaged from 50 μm (±25 μm) to 100 μm (±50 μm). Due to the low spatial resolution of the stimulation sites for Monkey F, we only used data from Monkey H. After this modification, the preferred spatial frequency range of the saccade MF angles closely matched the microstimulation results (B).

595
